# Digital-SMLM for precisely localizing emitters within the diffraction limit

**DOI:** 10.1515/nanoph-2023-0936

**Published:** 2024-06-06

**Authors:** Zhe Jia, Lingxiao Zhou, Haoyu Li, Jielei Ni, Danni Chen, Dongfei Guo, Bo Cao, Gang Liu, Guotao Liang, Qianwen Zhou, Xiaocong Yuan, Yanxiang Ni

**Affiliations:** Nanophotonics Research Center, Institute of Microscale Optoelectronics & State Key Laboratory of Radio Frequency Heterogeneous Integration, College of Physics and Optoelectronic Engineering & Key Laboratory of Optoelectronic Devices and Systems of Ministry of Education and Guangdong Province, 47890 Shenzhen University, Shenzhen 518060, China

**Keywords:** single molecule localization microscopy, deep learning, diffraction limit, sub-diffraction-limited spots, Digital-SMLM

## Abstract

Precisely pinpointing the positions of emitters within the diffraction limit is crucial for quantitative analysis or molecular mechanism investigation in biomedical research but has remained challenging unless exploiting single molecule localization microscopy (SMLM). Via integrating experimental spot dataset with deep learning, we develop a new approach, Digital-SMLM, to accurately predict emitter numbers and positions for sub-diffraction-limit spots with an accuracy of up to 98 % and a root mean square error as low as 14 nm. Digital-SMLM can accurately resolve two emitters at a close distance, e.g. 30 nm. Digital-SMLM outperforms Deep-STORM in predicting emitter numbers and positions for sub-diffraction-limited spots and recovering the ground truth distribution of molecules of interest. We have validated the generalization capability of Digital-SMLM using independent experimental data. Furthermore, Digital-SMLM complements SMLM by providing more accurate event number and precise emitter positions, enabling SMLM to closely approximate the natural state of high-density cellular structures.

## 1 Introduction

Simultaneous emission of two or more emitters at distance below the diffraction limit results in sub-diffraction limited spot, exceeding the resolution capability of conventional optical imaging system. Precisely localizing emitters at sub-diffraction limited displacements is crucial for quantification analysis and investigating molecular mechanisms in biomedical studies [1], [2], [3]. However, accomplishing this task has proven challenging, except when employing single molecule localization microscopy (SMLM) techniques, such as STORM and PALM. SMLM relies on specialized optical setup and imaging buffer to stochastically excite molecules of interest over a long acquisition time, providing molecule positions at high resolution. In this context, algorithms like compressed sensing [4] or MSSR [5] have been developed to complement STORM in fast imaging or, to some level, replace SMLM by providing high-resolution images. Despite achieving remarkable progress, these algorithms result in super-resolution images but fall short in accurately providing emitter counts and positions for sub-diffraction limited spot contributed by two or more emitters. This limitation makes them suboptimal for quantifying and pinpointing biological molecules within cellular ultrastracture or complex.

To address this issue, deep learning has been applied, achieving significant progress. Deep learning networks, particularly convolutional neural networks (CNNs), have been applied to break the diffraction limit and enhance qualities of microscopic images [6], [7], [8], as they can transform low-dimensional information from images into high-dimensional information through the convolution module with continuous overlap [9], [10], [11], [12]. For instance, a deep Fourier channel-based attention network DFCAN, its variant network DFGAN, and DL-SIM have demonstrated the capability to achieve super-resolution images at STED or SIM level from varied modalities of conventional imaging [13], [14]. These methods use end-to-end image generation to produce high-resolution images based on low-resolution images. ANNA-PALM, utilizing PALM images as training data, achieves reconstruction of high-quality super-resolution images using two orders of magnitude fewer frames than normally required without degrading the spatial resolution by generating adversarial networks [15]. Aiming to achieve fast STORM imaging, Deep-STORM, DeepLoco, and DECODE generated super-resolution images like spikes or outputted a set of coordinates with confidence higher than a certain threshold [16], [17], [18], [19], [20]. These algorithms have significantly advanced the development and application of SMLM. However, to accurately predict the exact number and precise positions of emitters at sub-diffraction limited distance(s) and resulting in sub-diffraction limited spots, new CCN algorithm and strategy are needed, as CNNs have the intrinsic inductive biases (IBs) of locality and scale-invariance [21], [22], [23], [24], making them effective in extracting local features and understanding the semantics in images.

In this study, we aimed to develop a method capable of determining number and positions for biological molecules within sub-diffraction-sized cellular ultrastructures or complex, such as receptor or other biological molecule cluster in cellular membrane. To this end, we adopted a new strategy by integrating a CNN with experiment-derived spot data, which encompass spot images from experiments along with corresponding emitter count and position(s) for each spot. Using this dataset, we trained a CNN algorithm, ResNet50 [21], to obtain both classification and regression models. When subjecting the testing set to the obtained models, we found the classification models achieved an accuracy of up to 98 % in determining emitter counts for sub-diffraction-limited spots while the regression models precisely pinpointed the emitters, achieving a root mean square (RMS) error as low as 14 nm for sub-diffraction-limited spots arising from two emitters at distance less than 60 nm. Noticeably, Digital-SMLM was capable to resolve two closely-locating emitters at a distance of 30 nm and outperformed deep-STORM in term of accurately predicting emitter numbers and positions for two-emitter sub-diffraction-limited spots. High accuracy of approximately 97 % was achieved when applying this method to imaging data from an independent experiment, confirming the generalization capability of this model. Digital-SMLM enhanced the accuracy of event number determination and emitter localizations for STORM imaging of biological ultrastructure, which complements STORM and allows it to approach the natural structure of high-density biological ultrastructures more closely.

## 2 Results

### 2.1 Strategy for precisely determining emitter numbers and positions for sub-diffraction-limited spots

To develop CNN models capable of accurately predicting emitter positions for sub-diffraction-limited spots with varying emitters, we needed to acquire a series of sub-diffraction-limited spots along with their ground truth emitter counts and positions to establish the training and testing sets. While simulated spots have been commonly used in previous reports [18], their emitter position is meaningless as they are regular Gaussian spots. In this study, we opted to acquire single molecule spots from experiments, as these spots possess a diverse array of features representing real emission spots. The corresponding emitter position of these single molecule spots can be determined by fitting the point spread function (PSF) to a Gaussian function. However, for sub-diffraction-limited spots with more than one emitter, obtaining a large amount of these spots and their emitter position information directly from imaging of molecules or sub-diffraction limit structures with a certain number of fluorescent emitters is challenging. Therefore, we summed the experiment-derived single-emitter spots to generate sub-diffraction-limited spots with two or a few emitters for training a deep learning model.

**Figure 1:**
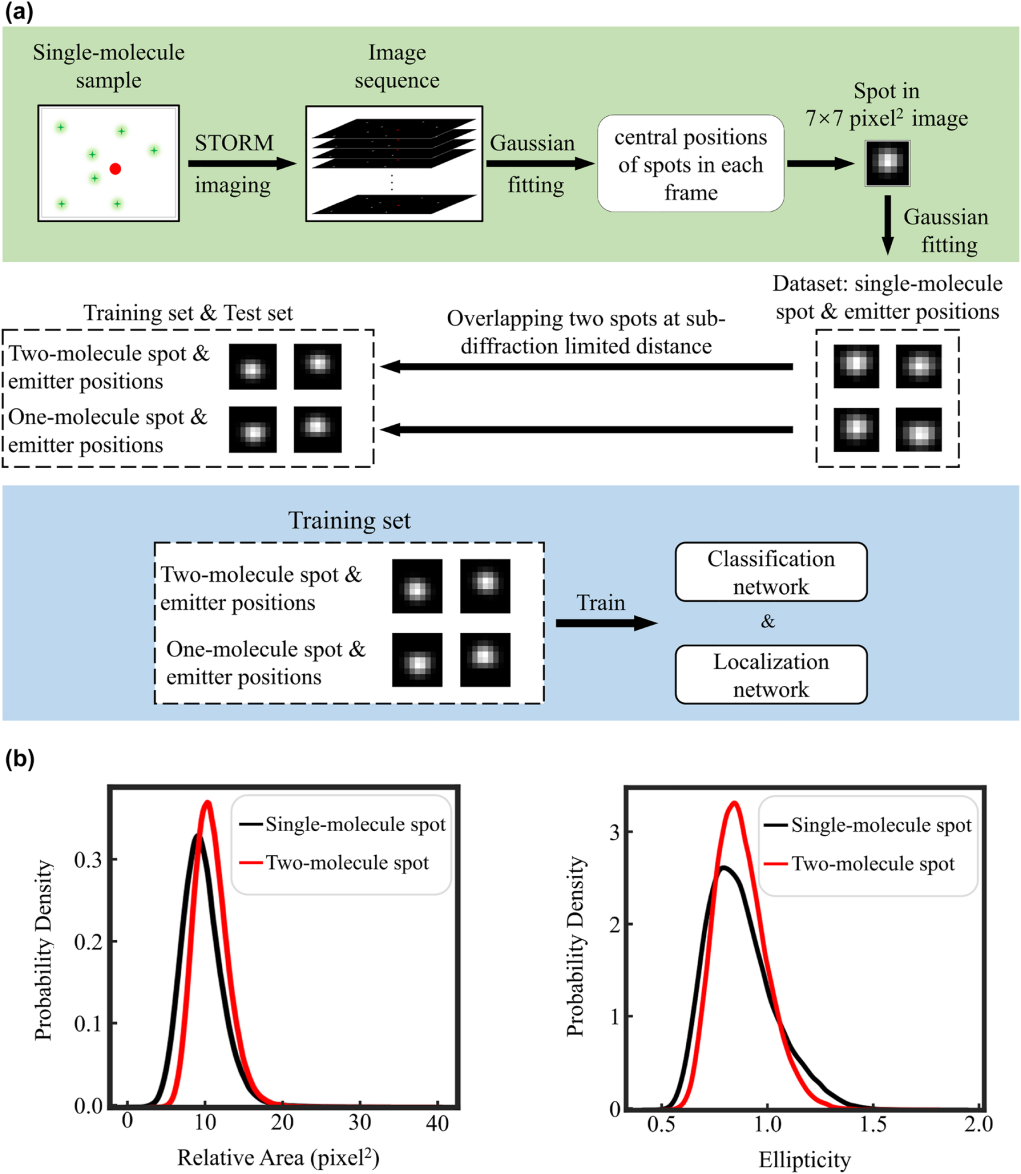
Strategy of Digital-SMLM and the characteristics of experiment-derived spots used. (a) Flowchart of Digital-SMLM strategy consists of three main parts. In the first part (marked with green background), we collected a series of 8000-frame image sequences in STORM imaging of single AF647 samples. We then localized the central position for each emission spot in each camera frame and performed segment processing for each spot via extending 3 pixels around its central position. Subsequently, we obtained a dataset of 7 × 7 pixel^2^ images, each of which contains one single-molecule emission spot, along with their corresponding emitter positions. In the second part (marked with white background), we randomly selected two single-molecule spots from the above dataset and merged them precisely according to their emitter positions at sub-diffraction limited displacement, which defined by less than 240 nm. In this way, we obtained a series of two-molecule sub-diffraction-limited spot as well as their corresponding emitter position coordinates. Eventually, we constructed a dataset containing single-molecule or two-molecule sub-diffraction limited spots along with their corresponding emitter counts and positions. In the third section (blue background), we trained a ResNet-50 residual network using the training set of single-molecule or two-molecule sub-diffraction limited spots to decode the emitter counts or positions, which is referred as classification network and regression network, respectively. (b) Probability density distribution of relative area and ellipticity for 14 thousands single-molecule (black lines) or two-molecule (red lines) sub-diffraction limited spots.

To ensure that the single-emitter spot images acquired are genuinely formed by single emitter activation, we performed STORM imaging of single molecule samples. We localized the potential position of each underlying emitter via Gaussian fitting (Figure 1a, green section, details see Method Section 4.2). To obtain sub-diffraction limited spot originating from two emitters that are at any sub-diffraction limited distance, we randomly picked two single-molecule spots and summed them after placing their emitter positions at sub-diffraction-limited displacement (Figure 1a, white section). Given the AF647 fluorescent molecules used in this study, the corresponding diffraction limit here is approximately 240 nm as per Abbe’s theory. Then, we utilized the resulting dataset to train the CNN algorithm, ResNet50, which generated models for classifying the two spot types and for regressing the positions of underlying emitters (Figure 1a, blue section).

Due to the minor displacements of less than 240 nm, statistical analysis indicated comparable sizes among one-emitter or two-emitter sub-diffraction-limited spots, totaling 150 thousand for each type (Figure 1b, left). Here spot sizes were evaluated via calculation of the relative area of each emission spot as

(1) $$\text{Relative}\;\;\text{area }=\;2\sigma_{x}\times 2\sigma_{y}$$

Here, *σ*_*x*_ or *σ*_*y*_ denote standard deviation of the widths of spots in *x*- or *y*-dimension, respectively.

Using the same batches of spots, we analyzed spot ellipticity, which was calculated as

(2) $$\text{Ellipiticity }=\;\frac{\sigma_{x}}{\sigma_{y}}$$

Here, *σ*_*x*_ or *σ*_*y*_ denote standard deviation of the widths of spots in *x*- or *y*-dimension, respectively.

The ellipticities were observed indistinguishable between the two types of spots (Figure 1b, right). Therefore, distinguishing one- or two-emitter sub-diffraction-limited spots solely based on their morphologic features is challenging, even more difficult to determine the underlying emitter position.

### 2.2 Training and verifying a CNN for predicting emitter numbers and positions of sub-diffraction limited spots

**Figure 2:**
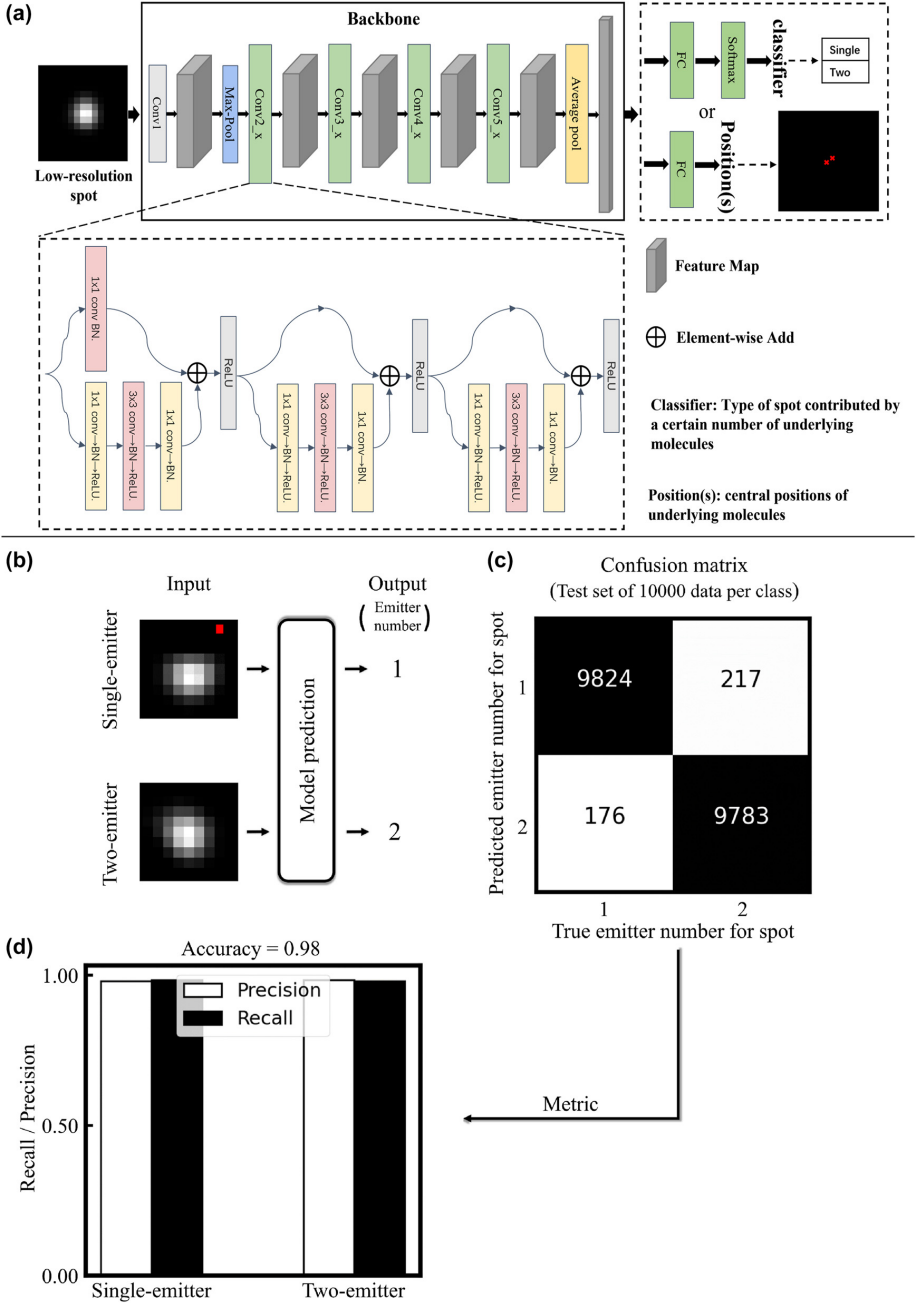
The process of training CNN networks and its performance in predicting emitter counts. (a) We applied ResNet-50 as a backbone and modified the output layer of the network to be function as a trainable classification network and a localization network. The output dimension of the localization network is determined by the number of emitter spots per image in the corresponding training set. ResNet-50 is composed of four residual blocks (light green), each of which consists of three jump connections, convolution, batch normalization and ReLU operations. The constant mapping constructed by the jump connections in the residual blocks effectively addresses the problem of network degradation by allowing a large amount of low-frequency information to bypass the redundant layers and allowing the neural network to learn high-frequency information more efficiently [21]. (b) We used 280 thousand images as training set to train a binary classification network for two types of images corresponding to single-emitter spot and double spots, and 20 thousand images as testing set. Scale bar, 120 nm. (c) Confusion matrix plotted based on the prediction of the classification network on the testing set, with the horizontal and vertical coordinates representing the number of true and predicted emitter number for spot, respectively. Scale bar, 120 nm. (d) Accuracy, recall rate and precision histograms. White bars show precision values for the varied spots, whereas black bars show recall rate.

Next, we will establish models for distinguishing sub-diffraction limited spots originating from one or two emitters and for accurately localizing their underlying emitters. We employed ResNet-50, which is composed of residuals and fuses low-dimensional and high-dimensional information of image, as the backbone network and trained it on the experiment-derived training set (Figure 2a, left and middle). In the training set, the input comprises 140 thousand images of one- or two-emitter sub-diffraction-limited spot, each image containing a single spot. For the classification network, the output consists of the ground truth emitter count for each spot, while the regression network’s output comprised the corresponding emitter positions (Figure 2a, right). Throughout training process, we flexibly adjusted the network’s output to match its intended function, aiming to accurately predict both emitter number and positions.

After completing the training process, we evaluated the performance of the established models using the testing data set. This dataset comprised 20 thousand spot images along with corresponding information on emitter counts and emitter positions. As shown in Figure 2b, two spots from the testing set were subjected to the classification network as an example and yielded a prediction of 1 or 2 as the emitter count, which perfectly matches the ground truth values. Likewise, predictions were achieved for the remaining spots in the testing dataset. These results allowed us to construct the confusion matrix that comprised true positive (TP), false positive (FP), false negative (FN), and true negative (TN) (Figure 2c). Utilizing this information, we calculated the precision, recall, and accuracy of spot classification with the following formulas:

(3) $$\text{Recall }=\;\frac{\text{TP}}{\text{TP }+\text{ FN}}$$

(4) $$\text{Precision }=\;\frac{\text{TP}}{\text{TP }+\text{ FP}}$$

(5) $$\text{Accuracy }=\;\frac{\text{TP }+\text{ TN}}{\text{TP }+\text{ FN }+\text{ FP }+\text{ TN}}$$

The results revealed that the classification network achieved a precision and recall of exceeding 97.8 % in distinguishing two-emitter spots from single-emitter spots, while the overall accuracy reached an impressive 98 % (Figure 2d).

**Figure 3:**
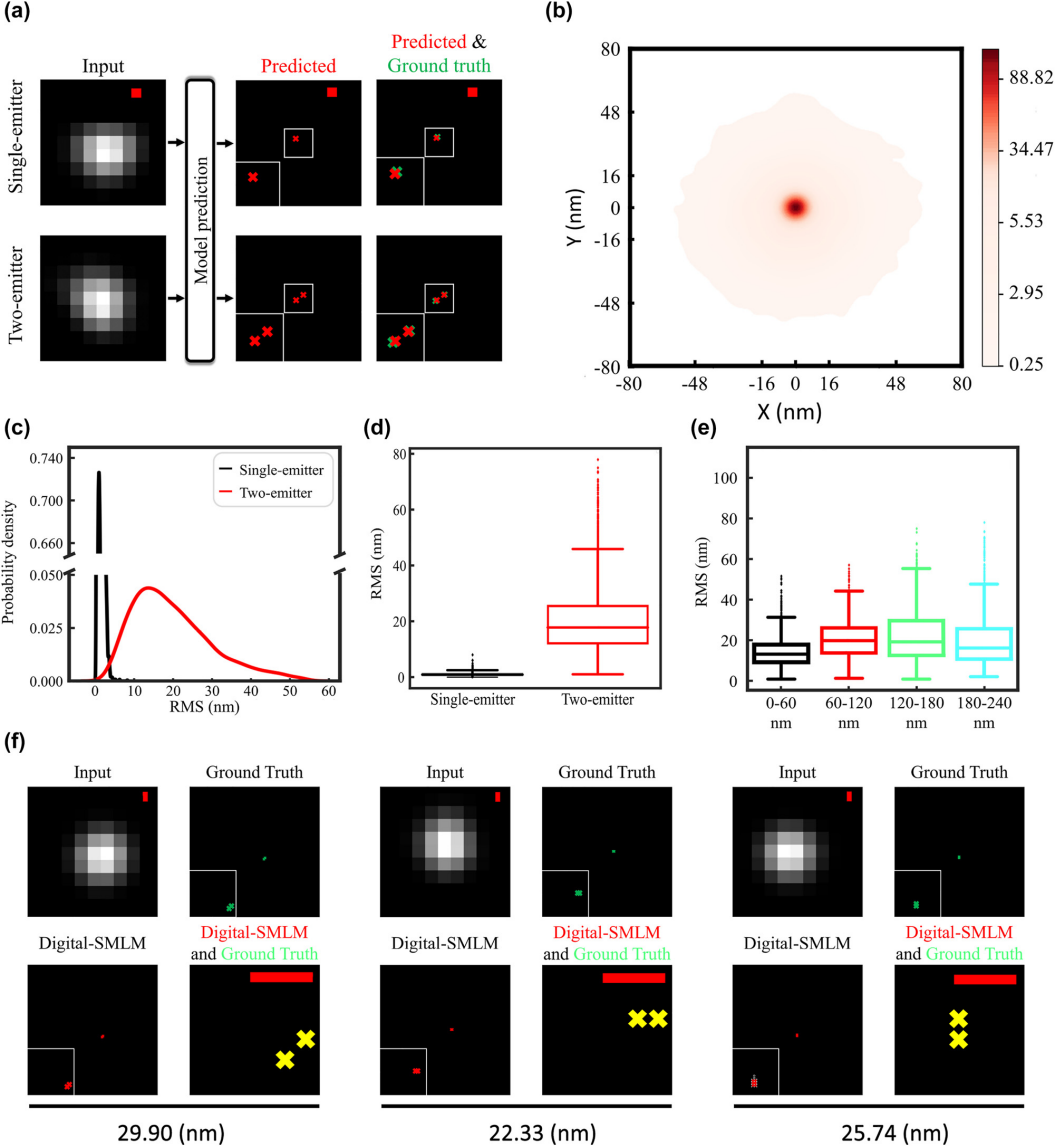
The performance in predicting emitter positions. (a) We used 280 thousand images as training set to train two localization networks, one emitter spot localization and two emitter spot localization, and each network was tested using 10 thousand images as a testing set. (b) For sub-diffraction-limited spots with two emitters, the ground truth positions of 20 thousand emitters are present at the origin of the coordinate system, while the predicted position of each emitter is accurately plotted in this system based on its relative coordinates to the corresponding ground truth. The heat map illustrates probability density of predictions at specific coordinates. The color bar ranging from white to red indicates probability density values from 0.2 to 112.2. (c–d) Each RMS value was calculated using formula (6) based on the predicted coordinates and the corresponding ground truth coordinates. Probability density distribution of RMSs for 10 thousand single-emitter spots (black line) or 10 thousand two-emitter (red line) spots are plotted (c). Based on these RMS values, the distribution box diagram are also plotted (d). (e) The RMSs for 10 thousand two-emitter spots in (c) were categorized into 4 groups according to the distance between two emitters that result in a sub-diffraction limit spot. The distribution box diagram of RMSs for the four groups (0–60 nm, 60–120 nm, 120–180 nm, and 180–240 nm) are separately plotted. (f) Predicted emitter positions for sub-diffraction-limited spot with two emitters at a distance between 20 and 30 nm. Emitter positions are plotted as green or red crosses based on coordinates from ground truth and Digital-SMLM prediction, respectively. The bottom right is a zoom-in image, with yellow crosses indicating complete match between the ground truth coordinate and the predicted coordinate in Digital-SMLM. Scale bare: 60 nm.

Then we applied the obtained regression network to predict the emitter positions for sub-diffraction-limited spots in the testing set. As shown in Figure 3a, the predicted positions (red crosses) for the two sub-diffraction-limited spots closely matched their corresponding ground truths (green crosses). Similarly, Digital-SMLM pinpointed potential emitter positions for the remaining spots in the testing set. To gain a comprehensive understanding of the emitter position prediction for two-emitter sub-diffraction-limited spots, we aligned all ground truth emitter positions at the origin of the coordinate system. Then, we plotted the predicted position for each emitter in the system according to its relative coordinates to the corresponding ground truth. The probability density heat map for predictions at different coordinates revealed a high abundance (red) of predictions within 10 nm proximity to the ground truths at the origin (Figure 3b), indicating accurate predictions.

To quantitatively evaluate the accuracy of emitter position prediction by this regression network, we calculated the root mean square (RMS) using the following formula:

(6) $$\text{RMS }=\;\sqrt{\frac{\underset{i}{\sum }{\;=\;1}^{I}\left({\left(x_{i}\;-\;{\bar{x}}_{i}\right)}^{2}\;+\;{\left(y_{i}\;-\;{\bar{y}}_{i}\right)}^{2}\right)}{2I}}$$

where ${\bar{x}}_{i}$ , ${\bar{y}}_{i}$ are the ground true coordinates of the emitter, *x*_*i*_, *y*_*i*_ are the centroid coordinates solved by the trained network, and I is the emitter count of the spot in a certain image.

In this way, we obtained RMSs for each predicted emitter position for 10 thousand single-emitter and 10 thousand two-emitter spots in the testing dataset (Figure 3c). Statistical analysis of all these RMS values demonstrated mean values of 20 nm for the two-emitter spots (Figure 3d). Based on the emitter displacements of these two-emitter spots, we categorized these spots into four groups, representing emitter distance range of 0–60 nm, 60–120 nm, 120–180 nm, and 180–240 nm. Our findings reveal that the mean RMS value can be as minimal as 14 nm for sub-diffraction limited spots with two emitters at a distance less than 60 nm (Figure 3e), demonstrating accurate predictions for emitter positions. Given that the recently-reported MSSR algorithm has demonstrated the capability to resolve two emitters at a distance of 40 nm [5], we carried out further testing to determine if Digital-SMLM could accurately predict positions for emitters at a distance less than 40 nm. Statistical analysis of RMSs for sub-diffraction-limited spots with two emitters at distance of 30 + 2 nm showed an averaged RMS of approximately 13.1 nm. As an example, the predicted positions of a sub-diffraction-limited spot with two emitters at a distance between 20 and 30 nm are displayed, with the predicted coordinates from Digital-SMLM closely aligning with the ground truth coordinates, indicated by the yellow crosses (Figure 3f). By exploiting this Digital-SMLM method, we can efficiently resolve two emitters at a sub-diffraction-limited distance of as close as 30 nm by accurately predicting their position based on their low-resolution spot image.

### 2.3 Digital-SMLM outperforms Deep-STORM in determining emitter numbers and positions and recovering the ground truth distribution of target molecules

**Figure 4:**
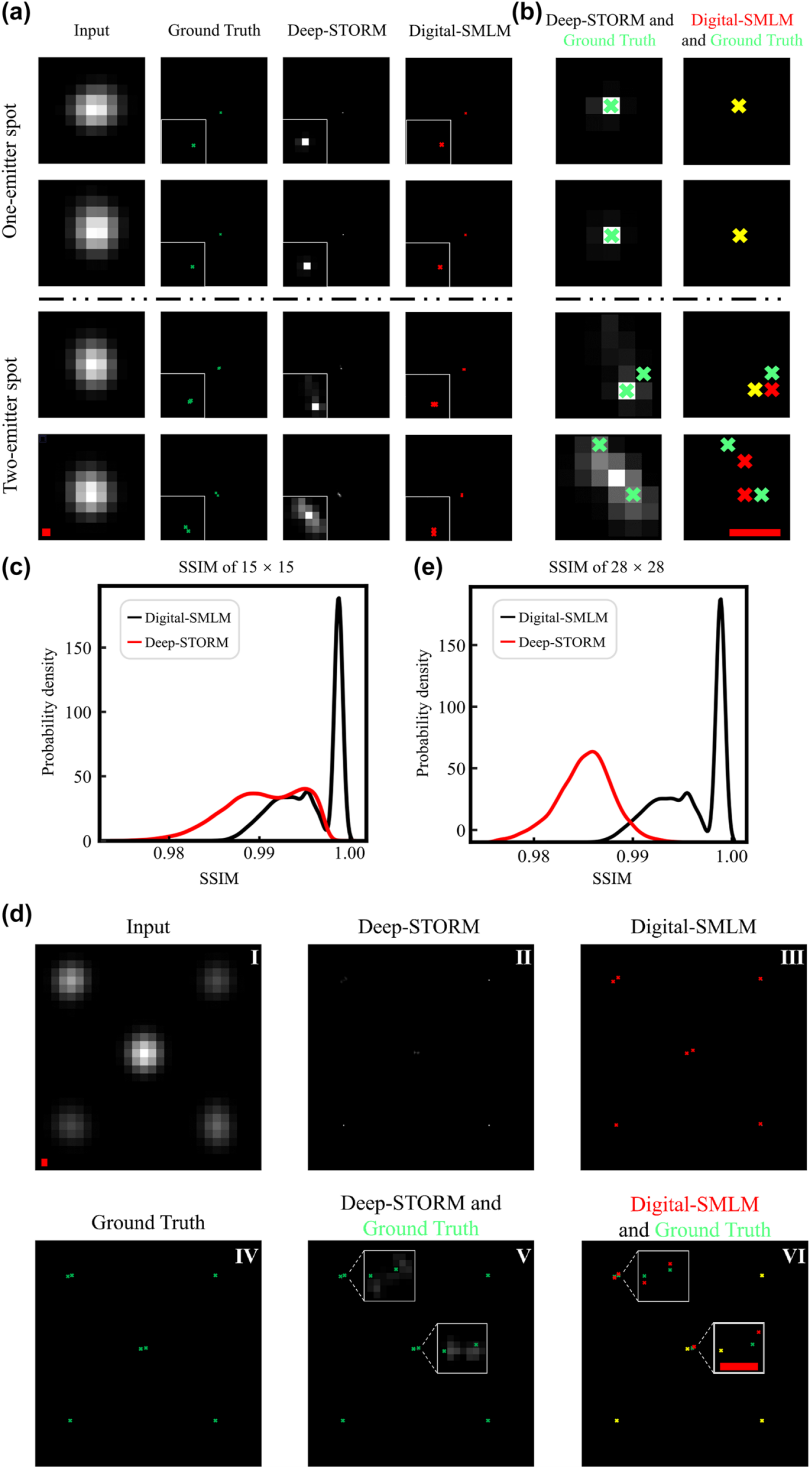
Compare Digital-SMLM with Deep-STORM in predicting emitter position for sub-diffraction limited spot. (a) Low-resolution 15 × 15 pixel^2^ images, each containing a one-emitter or two-emitter sub-diffraction-limited spot, were subjected Deep-STORM and Digital-SMLM, which were trained using the same training set consisting of spot images with dimensions of 15 × 15 pixel^2^. The ground truth emitter coordinates and the predicted emitter positions from Digital-SMLM are indicated as green crosses and red ones, respectively. The outputs from Deep-STORM are presented as spikes. Inserts show magnified images. Scale bar: 120 nm. (b) Magnified images of predicted spikes (grey grids) from Deep-STORM or predicted emitter coordinates (red crosses) from Digital-SMLM are overlaid with the corresponding magnified images of ground truth coordinates (green crosses). The yellow cross indicates a complete match between the ground truth coordinate and either the predicted coordinate in Digital-SMLM or the predicted spike in Deep-STORM. Scale bar: 60 nm. (c) For each low-resolution image, the spike image was obtained via plotted spike(s) at its emitter position(s) from ground truth or Digital-SMLM prediction, while the spike image could be directly obtained from Deep-STORM prediction. Then, corresponding heatmap images were obtained via convolving spikes with the same Gaussian kernel and applied to SSIM analysis, comparing the similarity between heatmap image for ground truth and heat map for either Deep-STORM or Digital-SMLM prediction. Similarly, SSIM value was obtained for each 15 × 15 pixel^2^ image in the testing set. Probability density distribution of SSIM values calculated for 10,000 single-emitter spot images (black line) or 10,000 two-emitter spot images (red line) are plotted. (d) Low-resolution 28 × 28 pixel^2^ image (*I*) was generated to consist of 3–5 experiment-derived sub-diffraction-limited spots arising from one or two emitter(s). This image was subjected to Deep-STORM, which had been trained on training set containing 28 × 28 pixel^2^ image, or cut to generate 15 × 15 pixel^2^ images, each containing a one-emitter or two-emitter sub-diffraction-limited spot. The resulting 15 × 15 pixel^2^ images subjected to Digital-SMLM, which had been trained on 15 × 15 pixel^2^ image dataset comprising of the same set of spots in the 28 × 28 pixel^2^ image training set for Deep-STORM. The ground truth emitter coordinates and the predicted emitter positions from Digital-SMLM are indicated as green crosses and red ones, respectively. The outputs from Deep-STORM are presented as spikes. Inserts show magnified images. Scale bare: 120 nm. (e) Heat map images were obtained for 28 × 28 pixel^2^ images using the same method as in (c). Probability density distribution of SSIM values calculated for 8000 heat maps from Digital-SMLM prediction (black line) or Deep-STORM prediction (red line), compared to those from ground truth, are plotted.

Next, we compared the capability of Digital-SMLM and Deep-STORM in localizing emitters for sub-diffraction-limited spot, considering that Deep-STORM is a fundamental algorithm in resolving overlapping spots [25], [26]. We started the comparison by training Deep-STORM models on the training dataset used in Figure 2 and subsequently applied the resulting models to the testing set from the same Figure. Unlike Digital-SMLM that explicitly predicts positions for emitters, Deep-STORM results in high-resolution spikes represented as pixels with varying grey levels. For most singles-molecule spots, either the peaks of spikes (grey pixels) from Deep-STORM or the predicted emitter positions from Digital-SMLM (red crosses) aligned well with the ground truth emitter positions (green crosses) (Figure 4a, upper rows). However, for a large portion of sub-diffraction limited spots with two emitters, the resulting spikes (grey pixels) from Deep-STORM displayed a single grey peak instead of two, which was clearly discerned in the inserted pictures (Figure 4a, lower rows). The discrepancy was further highlighted when magnified images from Deep-STORM or emitter coordinates from ground truths (green crosses) were overlaid in the left column (Figure 4b, lower rows). Meanwhile, predicted emitter positions (red crosses) for the same spots by Digital-SMLM closely aligned with the ground truths (green crosses), as demonstrated by the overlapping magnified images in the right column (Figure 4b, lower rows).

Due to different forms of prediction results from Deep-STORM and Digital-SMLM, a quantitative comparison necessitates the convolution of the predicted results from different methods, followed by evaluating the similarity between images from predictions and those from ground truths. To accomplish this, spikes were plotted according to emitter coordinates from ground truth or Digital-SMLM. We then convolved these spike images, as well as those directly from Deep-STORM, with the Gaussian kernel used to generate high-resolution heat map images in Deep-STORM. In this manner, 10 thousand heat map images were obtained from Deep-STORM, Digital-SMLM and ground truth for the low-resolution images in the testing set. For each testing image, its heat map from Deep-STORM and Digital-SMLM was then compared to heat map based on ground truth by calculating structure similarity index measure (SSIM). Statistical analysis of all obtained SSIM values revealed that, compared to heat maps based on ground truths, heat maps based on Digital-SMLM predictions exhibited a remarkably higher SSIM than those directly generated by Deep-STORM (Figure 4c). Here, we used SSIM to assess the comparison for several reasons. Firstly, both Digital-SMLM and Deep-STORM achieve labels based on spot images, but in different forms, without requiring additional information. Secondly, Digital-SMLM predicts coordinates directly, while the predicted spike in Deep-STORM can be interpreted as the network’s prediction of the probability of the emitter spot’s true position being present at each pixel. In this regard, it is implicated that both methods predict coordinates in different ways of presentation. Finally, a linear mapping of the output result of Digital-SMLM is all that is needed to obtain an image of the same nature as Deep-STORM from the output result.

We wonder whether the suboptimal performance of Deep-STORM is attributed to the presence of a single spot rather than multiple spots in each training set image. To address this, we generated dataset comprising relatively large images, each with dimensions of 28 × 28 pixel^2^ and containing three to five sub-diffraction-limited spots (Figure 4d, I). New models of Deep-STORM were directly trained using 80 % of these large images, whereas new models of Digital-SMLM were trained on 15 × 15 pixel^2^ images, each of which contains a sub-diffraction-limited spot from the large image. In regard to how to prepare the required small images for Digital-SMLM, the center information of each spot in each imaging frame, depicted as a 28 × 28 pixel^2^ image here, was firstly calculated by fitting to a two-dimensional Gaussian function. In cases where spot arising from emission of multiple emitters, Gaussian fitting gives an approximate center location rather than the true emitter position. Next, 11 × 11 pixel^2^ images are extracted via extending 5 pixels around the determined centers and enlarged to the required format of 15 × 15 pixel^2^ via zero fill. Subsequently, the models obtained from Deep-STORM or Digital-SMLM were applied to a testing set with images in the same form as those in the corresponding training set. For Deep-STORM models, the resulting spikes of a large number of two-emitter sub-diffraction-limited spots appeared as continuous grey pixels instead of two separated peaks, making it hardly to determine the putative positions for the emitters (Figure 4d, II). Meanwhile, for most of these two-emitter spots, Digital-SMLM can provide prediction for the two emitters.

Then, we merged the magnified super-resolution images from Deep-STORM (grey pixels) or emitter positions from Digital-SMLM prediction (red crosses) with the corresponding coordinates from ground truths (green crosses). We observed that the predicted emitter positions from Digital-SMLM were close to ground truth (Figure 4d, VI), whereas the spikes from Deep-STORM covered a relatively large area around the ground truth coordinates (Figure 4d, V). These results indicate a more accurate prediction by Digital-SMLM, also showcasing how Digital-SMLM could be applied to accurately pinpoint the emitters based on a single conventional imaging frame.

To make an unbiased comparison, we placed the predicted coordinates of emitter positions from Digital-SMLM back to the 28 × 28 pixel^2^ images. Then, we calculated SSIM to compare the similarity between each heat map image from Digital-SMLM or Deep-STORM predictions and the corresponding image from ground truths, as performed in Figure 4c. The SSIM analysis revealed that, compared to the image from Deep-STORM, images from Digital-SMLM were more similar to those from ground truth (Figure 4e). These results demonstrate that, in comparison to predictions from Deep-STORM, the predicted results from Digital-SMLM are closer to the ground truth, indicating that Digital-SMLM outperformed Deep-STORM in precisely predicting emitter number and positions for sub-diffraction-limited spots and recovering the ground truth distribution of molecules of interest.

### 2.4 Validating the generalization capability of Digital-SMLM with independent experimental data

**Figure 5:**
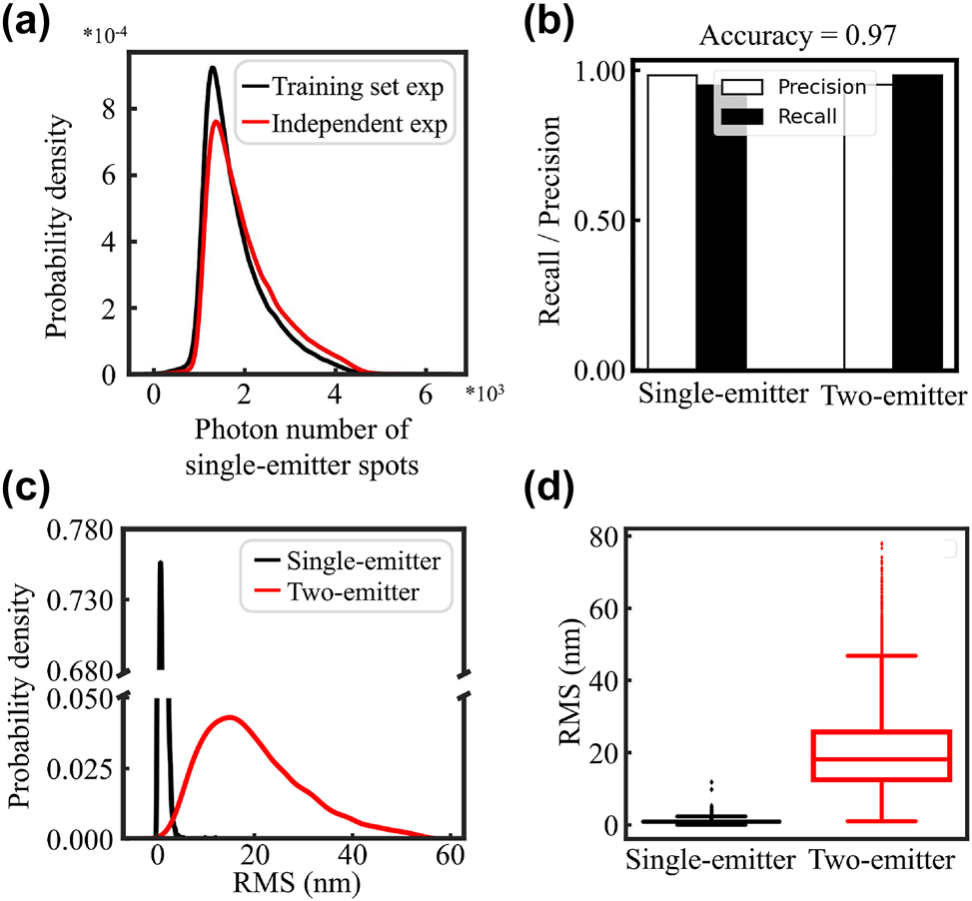
Validating the generalization capability of Digital-SMLM using dataset from independent experiment. (a) Photons number distribution compared between this independent experiment and the previous experiment data in Figure 4. (b) Accuracy, recall rate and precision histograms. White bars show the precision values of varied spots, whereas black bars show recall rate. (c) The root mean square (RMS) values were calculated using the coordinates of predicted and ground truth, the probability density curves were plotted as shown above. (d) The distribution box diagram of RMS.

To investigate the generalization capability of Digital-SMLM, we collected a new batch of single molecule spots from an independent STORM imaging experiment. Statistical analysis revealed that the distribution of spot photon numbers for the new batch is similar to that of the single-molecule spots within the training set used for the aforementioned models (Figure 5a). Next, we generated two-emitter sub-diffraction-limited spots via summing single-molecule spots and subjected 10 thousand images, each containing either a single- or two-emitter spots, to Digital-SMLM models obtained in Figure 2. Interestingly, we observed an accuracy approximately 97 % in classifying these two types of spots, with the high precision and recall shown in Figure 5b. Moreover, low RMS errors were observed in pinpointing emitters for the two-emitter sub-diffraction-limited spots from the independent experiment with a mean value of around 20 nm (Figure 5c and d). Therefore, these results implicated that Digital-SMLM can effectively determine emitter number and positions for sub-diffraction-limited spot from other experiments.

### 2.5 The extended Digital-SMLM for multi-emitter sub-diffraction-limited spots enables closely approaching to ground truth ultra-structures in STORM imaging

**Figure 6:**
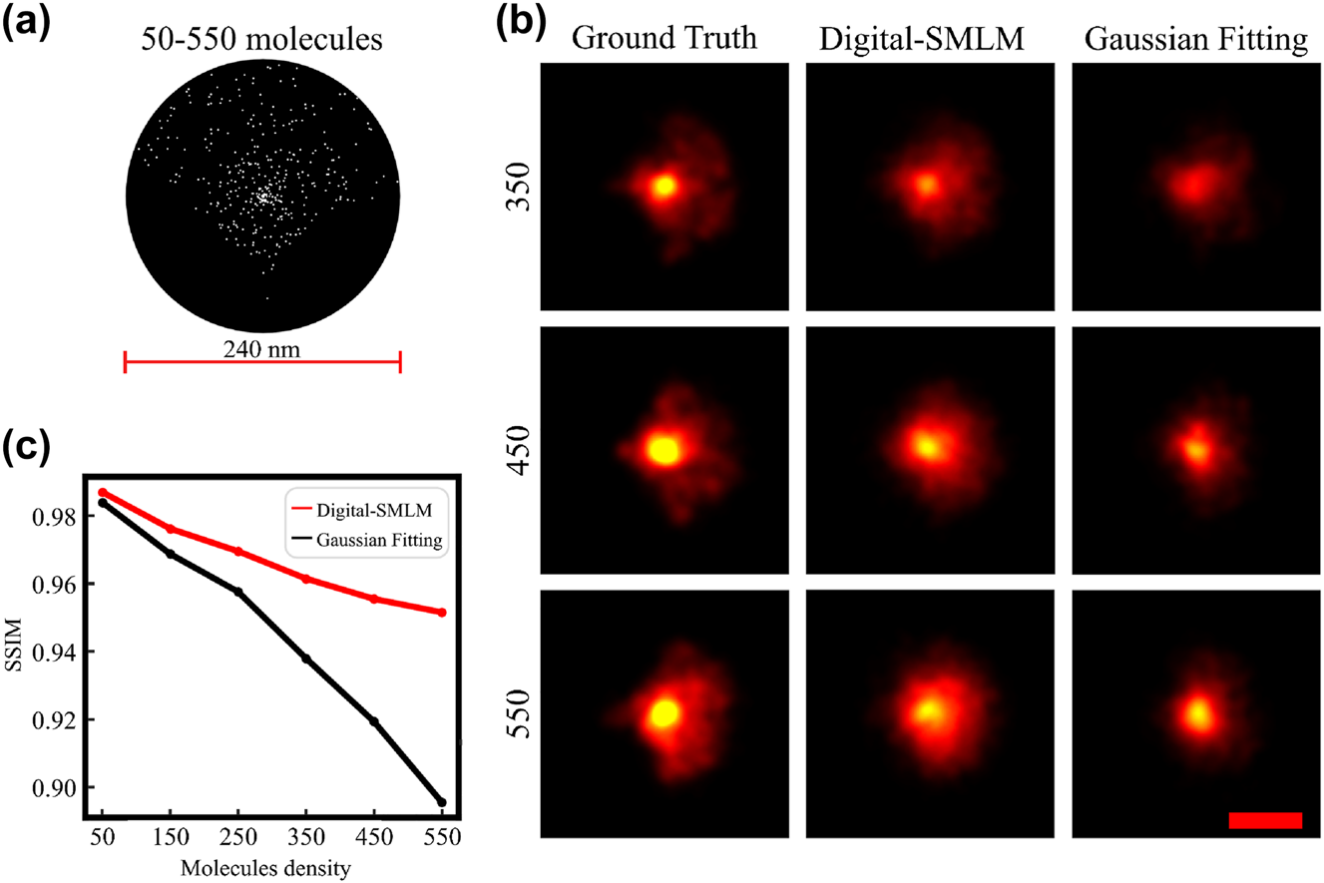
Digital-SMLM provides accurate predictions for localization counts and emitter positions for simulated STORM imaging of sub-diffraction-sized ultrastructures. (a) Schematic diagram of simulating the STORM imaging of non-uniform ultra-structures with higher densities in the center than in the periphery. Each 240 nm diffraction limited ultrastructure contains 50, 150, 250, 350, 450 or 550 molecules. (b) Super-resolution images for simulated STORM imaging of ultrastructures containing 350, 450 or 550 molecules were reconstructed based on ground truth coordinates, predicted coordinates from Digital-SMLM or single-molecule Gaussian fitting. The color ranging from black to red and then yellow indicates an increasing molecule density. Scale bar, 120 nm. (c) Structural similarity between images based on ground truth coordinates or images based on either Digital-SMLM prediction (red line) or single-molecule Gaussian fitting (black line) is calculated and plotted against the molecule density, which is represented as the number of molecules within the sub-diffraction limited region.

To further explore the potential application of Digital-SMLM, we simulated STORM imaging of biological sub-diffraction ultrastructures and subsequently applied Digital-SMLM to predict emitter positions for the obtained spots from the simulation. Aiming to mimic sub-diffraction-limited ultastructures in biological samples, we adopted an irregular sub-diffraction model that exhibited a non-uniform density pattern with molecule number ranging from 50 to 550 (Figure 6a). The simulation involved randomly selecting single molecules from the STORM experimental dataset and placing these molecules at the designated positions within the non-uniform ultrastructures. Through tracking the information of emission spots of each single molecule at different-numbered frames, we found that, in some frame of the simulated sequence, two or more molecules simultaneously emitted. This resulted in sub-diffraction-limited spots arising from two to four emitters, which are morphologically indistinguishable from single molecule spots. After simulating image sequence of ultra-structures, we applied Digital-SMLM to localize emitters for the obtained spots, including sub-diffraction-limited spots attributed to a few emitters.

Subsequently, we rendered the super resolution images for ultrastructures with various-densities based on spot localization from ground truth, Digital-SMLM prediction or Gaussian fitting. In Gaussian fitting, few emitter spots within the sub-diffraction limit due to small distances between emitter spots are treated as single molecules. As shown in Figure 6b, compared to super-resolution images based on coordinates from Gaussian fitting (right), the corresponding images from Digital-SMLM prediction (middle) appear more similar to those based on ground truth coordinates (left) for density of 350, 450 or 550 molecules within the ultrastructure. Consistently, SSIM analysis revealed that super-resolution structural patterns based on Digital-SMLM prediction are closers to those based on the ground truth coordinates (Figure 6c). Therefore, Digital-SMLM enables a close approximation to natural structures in STORM imaging of ultra-structures.

## 3 Conclusion and discussion

In this study, we have developed a novel CNN-based framework, Digital-SMLM, which focuses on addressing the challenging task of accurately pinpointing positions of emitters at sub-diffraction limited distance(s) in single imaging frame. For the first time, Digital-SMLM has achieved a remarkably low RMS error as low as 14 nm in pinpointing emitters for two-molecule sub-diffraction-limited spots, with high classification rates of up to 98 % in determining emitter counts for each spot (Figures 2 and 3).

To ensure training data containing spots variants in real experiments, we have implemented a unique approach for training set generation by deriving spot images from experimental STORM data, in contrast to previous deep learning algorithms that typically simulated training data based on point spread functions (PSFs) [16], [17], [18]. Aiming differently, Digital-SMLM inputs images containing one sub-diffraction limited spot arising from emission of one or a few emitters, whereas most previously-reported deep learning methods use larger-sized images containing multiple spots at various densities for training. During the training process, Digital-SMLM used the exact numbers and ground truth positions of emitters as training labels, while other methods produced super-resolution images like spikes or outputted a large number of probability-varied positions, exceeding the ground truth number of real emitters. Given that Digital-SMLM distinguishes itself from the previously-reported deep learning algorithms in many aspects of model training including the aforementioned one, we chose to compare it to Deep-STORM mainly because Deep-STORM is more comparable to Digital-SMLM in regard to the emitter distances than the other reported deep learning algorithms (Deep-STORM reaching emitter density of 9-emitters per square micrometer) and Deep-STORM is a fundamental deep learning algorithms with good reproducibility for comparison [16], [17], [18]. It turns out that Digital-SMLM outperformed Deep-STORM in precisely localizing emitters for sub-diffraction limited spots and recovering the ground truth distribution of target molecules (Figure 4). The observed suboptimal effect of Deep-STORM can be attributed to the fact that Digital-SMLM explicitly uses spot emitter positions as outputs of training set, while the training set outputs of Deep-STORM consist of high-resolution graphs, which include information of approximate emitter positions instead of the accurate emitter coordinates. It is worth note that recently-developed methods for generating high-resolution images, such as MSSR [5], has reached a promising spatial resolution of 40 nm, capable of resolving two emitters at 40 nm space despite providing no accurate emitter positions. Digital-SMLM can accurately pinpoint emitters at a distance of ∼30 nm with an averaged RMS of 13.1 nm based on their low-resolution sub-diffraction-limited spot (Figure 3c).

In STORM imaging of high-density biological ultrastructures, two or even more fluorescent molecules within sub-diffraction limited region may be activated simultaneously in certain frames of the imaging sequence, an occurrence that is theoretically rare but indicated by previous study [27], [28] or STORM imaging simulation using single molecules from real experiments (Figure 6). Given Digital-SMLM’s capability to accurately provide both emitter counts and positions for sub-diffraction-limited spots, it can effectively complement STORM in imaging cellular ultrastructures that consist of a high abundance of biological molecules within diffraction limit. Moreover, the distinct capability of Digital-SMLM provides a direct foundation for performing quantification at high resolution for low-abundance molecules in biological samples without the need for resorting to SMLM in future studies. Despite these advantages, Digital-SMLM is more suitable for sub-diffraction-sized cellular compartments or complex rather than larger structures. In our upcoming research, we aim to train additional models so that Digital-SMLM can predict emitter counts and positions for emission spots/patches with emitters at distance larger than diffraction limit, thereby extending its applicability range.

One limitation of this current manuscript is being unable to test the model’s performance on real cellular data. Verification of Digital-SMLM’s performance on real cellular data requires ground truth positions of the emitters, which are at sub-diffraction limited distance and simultaneously emit to generate sub-diffraction spot. However, obtaining such experimental data for verification, even using STORM imaging, is currently challenging. To make the simulated imaging closer to the real imaging, we chose to simulate STORM imaging of ultrastructure using experimental data rather than simply placing PSFs to form the structures (Figure 6). Noticeably, the experimental data used for the simulation contain the real blinking information of fluorophore in STORM imaging as well as a wide range of spot variants in real experiments.

Since various noise conditions are often encountered in practical imaging scenarios, understanding the potential impacts of different noise is important for evaluating the reliability and practical utility of Digital-SMLM. In this study, we had chosen to generate a training dataset based on “real” experiment data with varied noises rather than via simulation using PSFs with additional noise. In the training process or practical utility of Digital-SMLM, the images are applied only after using a band-pass filter that suppresses pixel noise while retaining necessary information. In this way, the impacts of various noise conditions on the model’s performance could be minimized. Consistently, testing set from an independent experiment achieved relatively high prediction accuracy (0.97) and low localization error (∼20 nm) (revised Figure 5). Systematic investigation of the performance of our digital-SMLM under different noise sources and different signal to noise ratio will be perform in the near future.

## 4 Methods

### 4.1 STORM experiments for collecting single molecule spots

To prepare single-molecule samples for STORM imaging, coverslips were cleaned by sonication and coated with 0.1 % gelatin. Fluorescent microspheres (F8810, Thermo Fisher) of 200 nm in diameter were fixed on the gelatin-coated coverslips with freshly-prepared 4 % paraformaldehyde for around 10 min at room temperature, so that they could be used as fiducial markers for sample drift correction in *x*–*y* plane during image acquisition. After being washed in PBS for multiple times, the glasses with beads were then incubated for 30 min with 1 μM oligonucleotides. Oligonucleotide was conjugated to an AF647 at its 5′ end and to biotin at its 3′ end, to allow for nonspecific association between biotin and gelatin, leading to immobilization of sparse oligonucleotides on the glass surface. Samples were subjected to STORM imaging using a customized Olympus IX-71 inverted microscope construction as previous reports [29], [30] with Numerical Aperture (NA) of 1.4, a magnification factor of 20, and 160 nm per pixel. Samples imaged were imbedded in widely-used imaging buffer [31], [32], [33]containing 50 mM Tris (pH 8.0), 10 mM NaCl, 1 % *β*-mercaptoethanol (v/v),10 % glucose (w/v), 0.5 mg/mL glucose oxidase (G2133, Sigma), and 40 ug/mL catalase (C30, Sigma). In most cases unless otherwise indicated, 8000-frame sequential images were collected at 33 Hz for each field of view for 8000 frames.

### 4.2 Establishment of single molecule spot dataset

The localization data with drift correction in Thunderstorm were collected from point-like objects, each of which appeared as a cluster of multiple localizations in single-molecule library samples. For each molecule, the photon distribution of the spots over 7 × 7 pixels and over 8000 frames were extracted from experiment, so that the blinking characteristics and photon statistics in simulation are close to experimental conditions. Consequently, we established a single-AF647 dataset with the temporal and position information of thousands of individual AF647 molecules from STORM imaging.

For raw image analysis, the localization of each single-molecule spot was determined via fitting to a two-dimensional Gaussian function. The two-dimensional Gaussian function represents the photon intensity distribution of a single emitter imaged by a microscope. The intensity profile *I*(*x*, *y*) of a spot at position (*x*, *y*) can be described by the formula:

(7) $$I\left(x,y\right)\;=\;I_{0}\exp\left(-\frac{{\left(x-x_{0}\right)}^{2}+{\left(y-y_{0}\right)}^{2}}{2\sigma^{2}}\right)$$

where *I*_0_ is the peak intensity, (*x*_0_, *y*_0_) is the centroid of the spot, and *σ* is the standard deviation related to the width of the Gaussian function. For each localization, its photon number was calculated as the sum of photons over the pixels of the corresponding spot. The size of the spot was calculated as the area (*πw*_*x*_ *w*_*y*_) of the ellipse whose short and long axes equal to the fitted widths (*w*_*x*_ and *w*_*y*_) of a two-dimensional Gaussian function:

(8) $$G\left(x,y\right)\;=\;h\;\exp\left(-\frac{{\left(x-x_{0}\right)}^{2}}{2{w_{x}}^{2}}-\frac{{\left(y-y_{0}\right)}^{2}}{2{w_{y}}^{2}}\right)$$

The ellipticity of the spot was calculated as ratio of the fitted widths *w*_*x*_/*w*_*y*_.

### 4.3 Derivation of multi-emitter spots from experimental single-molecule spots for generating the training data

To generate 2–4 emitter sub-diffraction-limited spots for the training set, we created a background image with a pixel size of 1 nm. Then, we drew a circular area with a diameter of 240 nm, corresponding to the diffraction limit in regard to the application of AF647 in this study. Within the designed area, we randomly scattered points to generate 2 million coordinates, which ensures that the distance between any two coordinates is not more than 240 nm. To generate a spot arising from co-emission of 2–4 emitters, we superimposed the corresponding number of single-molecule spot images from our single-molecule experimental database, and pinpoint images onto these randomly selected coordinates using the Gaussian-fitted emitter coordinates of single-molecule spots as anchor points. In this way, the ground truth emitter positions for the multi-emitter spots are available independent of prior or any additional information, since the emitter position of the single-molecule spot for the superimposition is readily determined via the two-dimensional Gaussian fitting. Consequently, the emitter distances of multi-emitter spots derived from single-molecule experimental spots are less than the diffraction limit.

### 4.4 Molecular spot classification and localization model

Theoretically, object detection algorithms are the most straightforward solution to this kind of problem. However, since the dataset we used contains highly-overlaid emitter spots, this makes the methods using object detection algorithms inappropriate for this type of problem. Therefore, we approach the problem from another perspective by converting the recognition of light spots into a two-step process of classification and regression localization. Such a treatment can effectively avoid the problem of difficult detection of highly overlapping targets in target detection, while the classification-based and regression-based approaches have achieved impressive results in many computer vision tasks [10], [11], [21], [34]–[40]. Therefore, as shown in Figure 2a, we trained two types of networks for classification and regression localization, respectively. The purpose of the classification network is to determine the number of emitter spots on each image in the dataset, which helps determine the number of coordinates to be returned. The output of the classification network will then be used to select the coordinates to be solved for using the corresponding regression localization network. To achieve this, we have fine-tuned the existing backbone network. In our approach, we use ResNet-50, a deep neural network developed by Kaiming He and his team at Microsoft Research Asia, as the backbone to extract features from the images (Figure 2a). ResNet introduced shortcut or skip connections that fuse low-dimensional information with high-dimensional information. This effectively solves the problems of network degradation and gradient dispersion as the neural network deepens, as well as the problem that as the network deepens, the low-dimensional information of the image is continually lost. In our improved network, the output layer is designed according to the class of the task as well as the number of light spots. As shown in Figure 2a, the output layer of the classification network consists of a fully-connected layer and a SoftMax activation function for classification, while the output layer of the localization network is reduced from a fully-connected layer to a direct output coordinate. Therefore, we need to train the localization network that can locate different number of emitter spots separately. Although this process may be laborious, this can accurately locate the precise coordinates of each emitter spot in images with different numbers of emitter spots, and this practice is a huge improvement in localization accuracy.

### 4.5 Loss function and training

For the classification task in the first stage mentioned above, we use cross-entropy as a loss function and our loss function is as follows:

(9) $${\text{L}}_{\text{class}}\;=\;\underset{i}{\sum }{\;=\;1}^{n}p\left(x_{i}\right)\text{log}\left(q\left(x_{i}\right)\right)$$

where *p*(*x*_*i*_) is the true value and $q\left(x_{i}\right)$ is the network prediction. For the regression localization network, we use the mean square error as the loss function. The great advantage of the MSE loss function is that it makes the mean of the final localization error smaller, as follows:

(10) $$L_{\text{position}}\;=\;\frac{1}{2I}\underset{i}{\sum }{\;=\;1}^{I}\left({\left(x_{i}\;-\;{\bar{x}}_{i}\right)}^{2}\;+\;{\left(y_{i}\;-\;{\bar{y}}_{i}\right)}^{2}\right)$$

where ${\bar{x}}_{i}$ , ${\bar{y}}_{i}$ are the true center of mass coordinates and *x*_*i*_, *y*_*i*_ are the center of mass coordinates of the spot predicted by the network.

To maximize the performance of the network, we trained approximately 400 epochs on the training set, used the Adam optimizer throughout the training process, added an *L*_2_ parametric penalty to prevent overfitting, and set the hyperparameter to 0.01. We set the batch size to 64, used the training learning rate scheduler and used Cosine annealing to decay the learning rate from an initial 1*e* ^−5^ to 1*e* ^−8^ in 400 epochs. Our experiments have shown that a low initial learning rate setting and a large decay in the learning rate during training improves the performance of the network, as the input to the network is a single channel image with 15 × 15 pixels of overly simple information. The hardware specifications of the PCs used for training and testing in the above method are Intel 10-core, 12-thread, 256 GB of RAM, 2.2 GHz cpu and NVIDIA Tesla V100S GPU (32 GB RAM).

## Supplementary document

See Supplementary 1 for supporting content.

## Supplementary Material

Supplementary Material Details
