# Supplementary material for: Digital-SMLM for precisely localizing emitters within the diffraction limit
Source: Nanophotonics. 2024 Jun 6;13(19):3647–61. doi: 10.1515/nanoph-2023-0936 (PMC11465993; doi:10.1515/nanoph-2023-0936)
Supplement: Supplementary file 1 — Supplementary Material Details [file j_nanoph-2023-0936_suppl_001.docx]

Digital-SMLM for Precisely Localizing Emitters within the Diffraction Limit: supplemental document

Before comparing the capability of Digital-SMLM and Deep-STORM in localizing emitters for sub-diffraction-limited spot, we established Deep-STORM in our laboratory and utilized it to analyze previously-reported images, in which simulated spots overlap and form irregular emission patches (Extended Fig. 1, left). The obtained spikes from Deep-STORM (Extended Fig. 1, right) are consistent with the results in previous report [1], validating the performance of Deep-STORM in our laboratory for resolving previously-reported overlapping spots. **
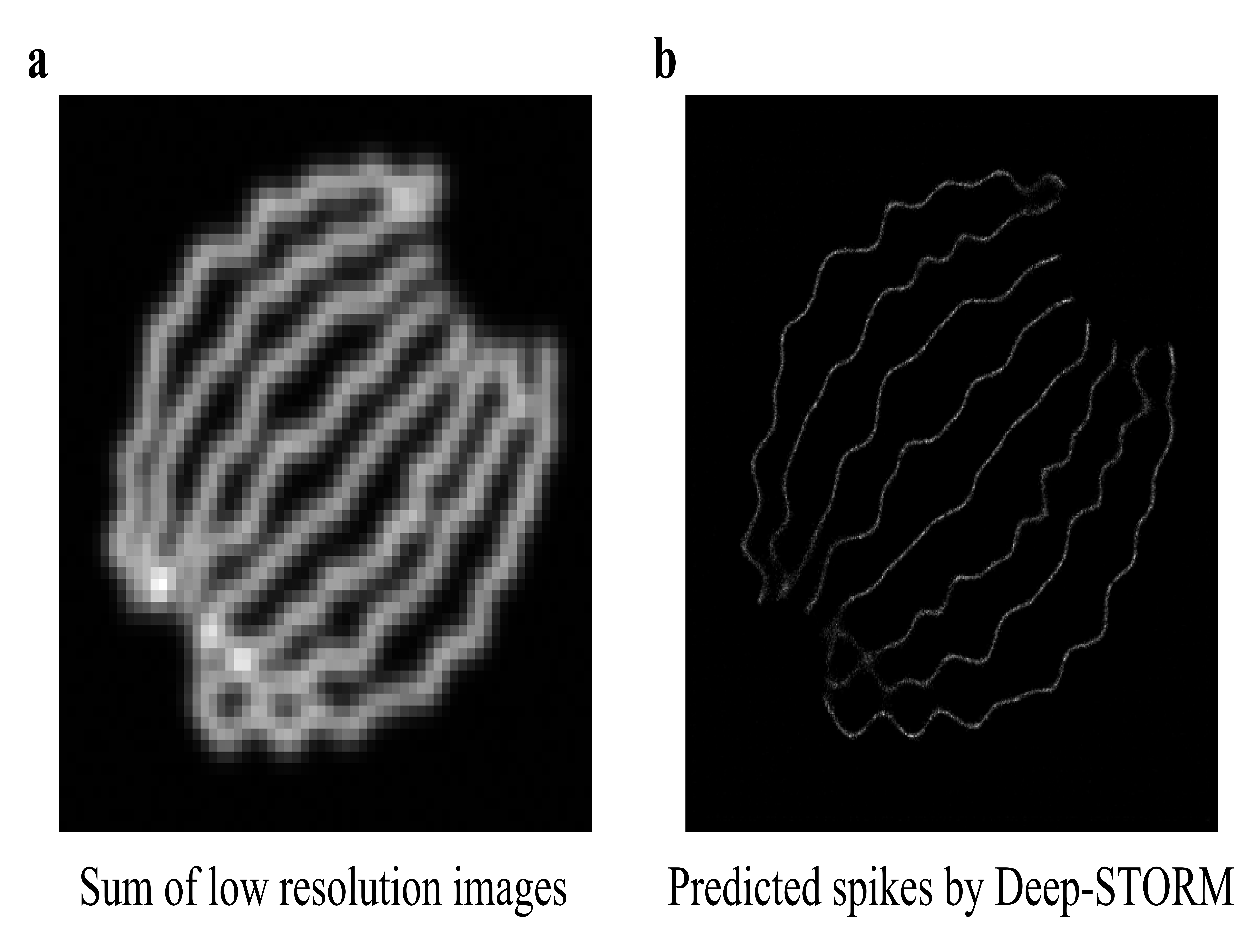
**

**Extended Fig. 1. The result of Deep-STORM replication.** (a) Superposition of low resolution image frames. (b) Deep-STORM was used to predict low-resolution image frames, and frame superposition was performed on the obtained spike images.

Given the remarkable success of Digital-SMLM in predicting emitter counts and positions for sub-diffraction-limited spots with two emitters, our next step was to extend the prediction capabilities of this method for sub-diffraction-limited spots arising from multiple emitters. Follow the same method as before for training the network, we obtained models for classifying sub-diffraction-limited spots arising from one to four emitters or regression models for pinpointing their corresponding emitters. Subsequently, these models were applied to a testing set comprising four different spot types, each containing10,000 images. Based on the data presented in the confusion matrix (**Extended Fig. 2a**), we achieved an overall accuracy of approximately 83% in classifying these four spot types, with precision and recall values depicted in **Extended Figure 2b**. Notably, probability density values of RMS errors remained relatively low, even for three- or four-emitter sub-diffraction-limited spots (**Extended Fig. 2c**), with median value of 20 nm, 30 nm, and 34 nm for two-, three-, and four-emitter spot, respectively(**Extended Fig. 2d**).

**
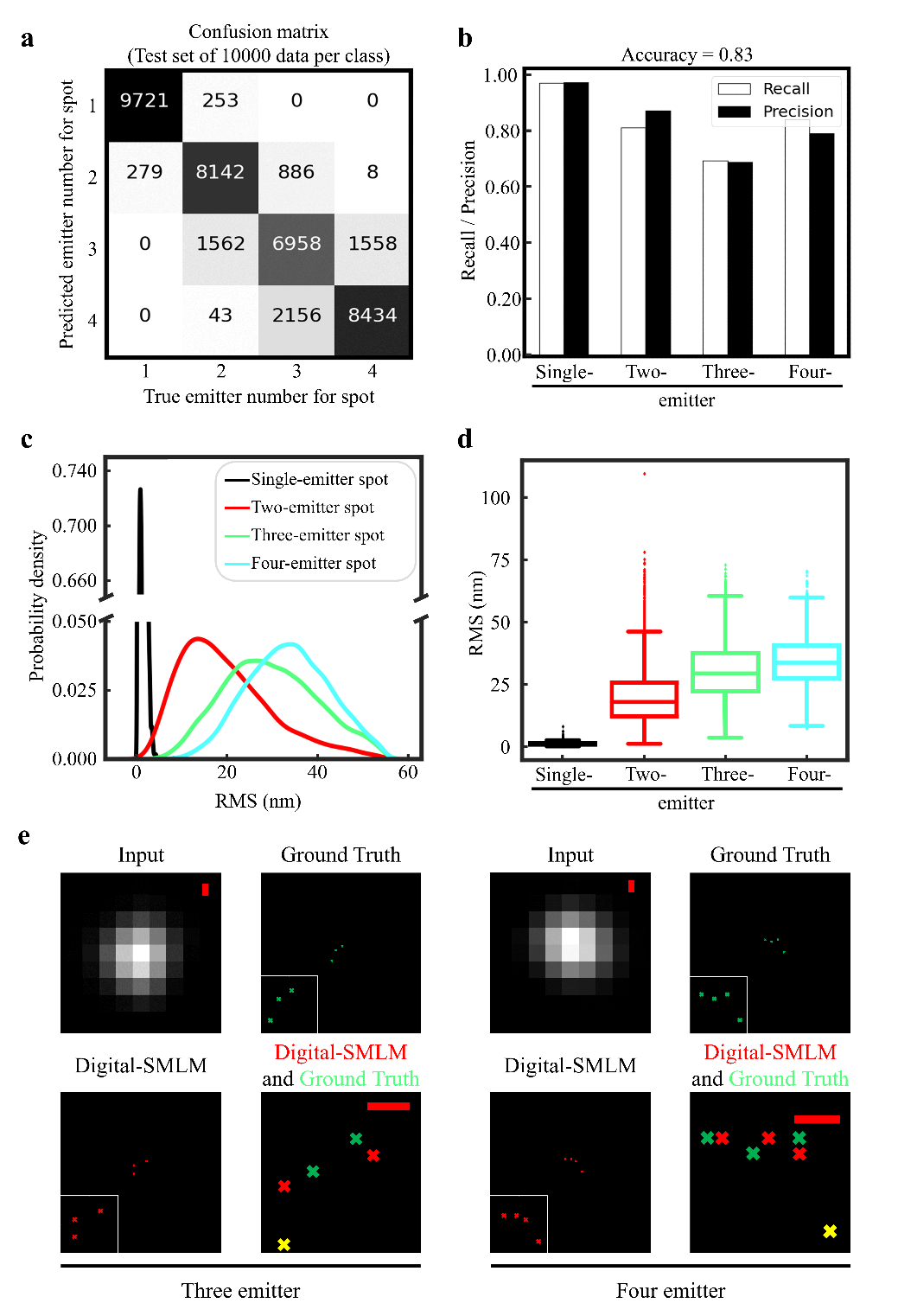
**

**Extended Fig. 2. Four kinds of emitter spot classification and localization effect.** (a) Confusion matrix plotted based on the prediction of the classification network on the test set, with the horizontal and vertical coordinates representing the number of true and predicted emitter number for spot, respectively. (b) Accuracy, recall and precision histograms. White bars show precision values for the varied spots, whereas black bars show recall. (c) The root mean square (RMS) values were calculated using the predicted coordinates and ground truth, and the probability density curves were plotted as shown in Fig. (d) The distribution box diagram of RMS is shown in Fig. (e) Extended Fig. 2e Emitter prediction results of three- and four-emitter subdiffraction limited spots by Digital-SMLM. Emitter positions are plotted as green or red crosses based on coordinates from Ground truth and Digital-SMLM prediction, respectively. The Bottom right is a zoom-in image, with yellow crosses indicating complete match between the Ground Truth coordinate and the predicted coordinate in Digital-SMLM. Scale bare: 60 nm

Our network does exist cumulative error caused by the two-stage method. Since the localization network needs to know the number of spots in advance, the classification network will predict the number of molecules. Therefore, it is necessary to consider the situation where the localization network is told the wrong number of molecules, i.e., the classification network makes wrong predictions. However, subjectively speaking, considering that the classification accuracy of the binary classification network is 98%, the situation where the localization network is told the wrong number of molecules is relatively rare. However, the classification accuracy of the four-class network is 83%, so the probability of the above situation is relatively high. We have designed the following experiments to verify the impact of cumulative error caused by the two-stage method. We tested in two scenarios. First, we assumed that the classification accuracy of the classification network for binary and four-class classification is completely correct. The localization network then located the data in the test set based on the completely correct classification results. The obtained results were calculated for SSIM with the Ground truth using the same method as in Figure 4c of the main text and statistically analyzed. We call the results obtained by this method the results using prior knowledge. Secondly, we used the prediction results of the classification network as the criterion, and the localization network located the data in the test set based on the classification results with some errors. The obtained results were then calculated for SSIM with the Ground truth and statistically analyzed. We refer to the results obtained through this method as those obtained without using prior knowledge. The results of these two scenarios were then plotted into a probability density histogram as shown in **Extended Figure 3**. As can be seen from the figure, in the results of the binary classification scheme, the red line almost completely overlaps with the black line, so the decrease in accuracy caused by cumulative error is not obvious. Although the red line and the black line cannot be completely overlapped in the four-class task, the overlapping part still accounts for a large proportion. Therefore, to a certain extent, it also shows that the effect of cumulative error is not as large as we expected, especially in the four types of tasks with low classification accuracy. However, we also acknowledge that cumulative error will lead to the loss of accuracy and the weakening of network robustness, and we hope to further improve this in future work.


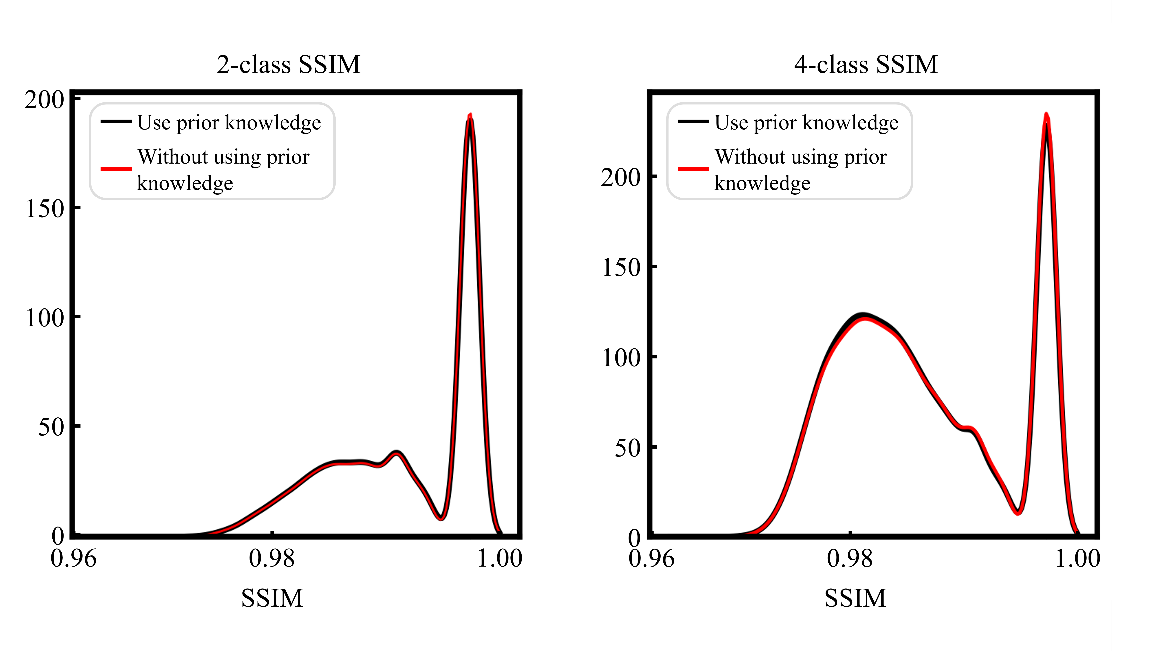


**Extended Fig. 3. The influence of network cumulative error on network robustness.** The two figures on the left and right respectively demonstrate the probability density statistics of the SSIM between the predicted results with and without the use of categorical prior knowledge and the Ground truth for the test set data in both binary and four-class classification tasks. In the figures, the red line represents the scenario where categorical prior knowledge is not utilized, whereas the black line represents the outcomes obtained when categorical prior knowledge is employed.

Considering that the output of the network may be biased in further applications of the two-stage model, we also tried to train a network capable of performing both classification and localization tasks, by which we hope to enhance the consistency of the network for feature extraction. As shown in **Extended Figure 4a**, we connected two output headers (classification header and localization header) behind the backbone of the network. Specifically, the classification header is used to filter out the incorrectly predicted coordinates in the localization header, e.g., when the classification header predicts a single-emitter spot, we only need to take out the first coordinate predicted by the localization header. This approach is inspired by previous excellent work that also filtered out coordinates with low confidence to obtain the final prediction [2]. Subsequently, the trained model was applied to the same test set as in the main text section containing two different emitter spot types, each containing 10,000 images. Based on the data in the confusion matrix (**Extended Fig. 4b**), the overall accuracy of our classification is about 98%, and the precision and recall values are shown in **Extended Figure 4c**. It is worth noting that the localization accuracy is reduced compared to the two-stage approach (**Extended Fig. 4d**), with average RMS errors of 10 nm and 41 nm for single and two-emitter spots, respectively (**Extended** **Fig. 4e**). We believe that it is also normal for this phenomenon to occur, and that the two-stage model achieves the realization of the task through multiple models, with the ability of each model to learn to extract features depending on the tuning of the parameters for the realization of its own task. Whereas a single-stage integrated model does have an increase in efficiency, it will inevitably result in a loss of accuracy as the models are expect ed to have the ability to extract different features for multiple tasks. This phenomenon has similar cases in the work in the field of target detection, for example, the two-stage models, led by the RCNN series of models, tend to be more accurate than the single-stage models, led by YOLO [3-5], but the two-stage models tend to be inferior to the single-stage models in terms of speed.

We also explored the convergence of the neural network model during training. For this purpose, we plotted the **Extended Figure 5**. which visualizes the gradual decrease in the value of the network loss function as the training time progresses. The horizontal axis represents the training time, while the vertical axis reflects the value of the network loss function. It can be noticed that the loss function value is minimized at about 2.5h of training, and the loss function value tends to stabilize after 4 hours. We also counted the relevant technical metrics and listed them in the **Extended Table 1**. The table lists key information such as the number of network parameters, computational complexity and inference time. These metrics not only help us understand the scale and complexity of the model, but also provide important references for subsequent model optimization and performance improvement. The hardware specifications of the PCs used for training and testing in the above method are Intel 10-core, 12-thread,256GB of RAM, 2.2GHz cpu and NVIDIA Tesla V100S GPU (32GB RAM).


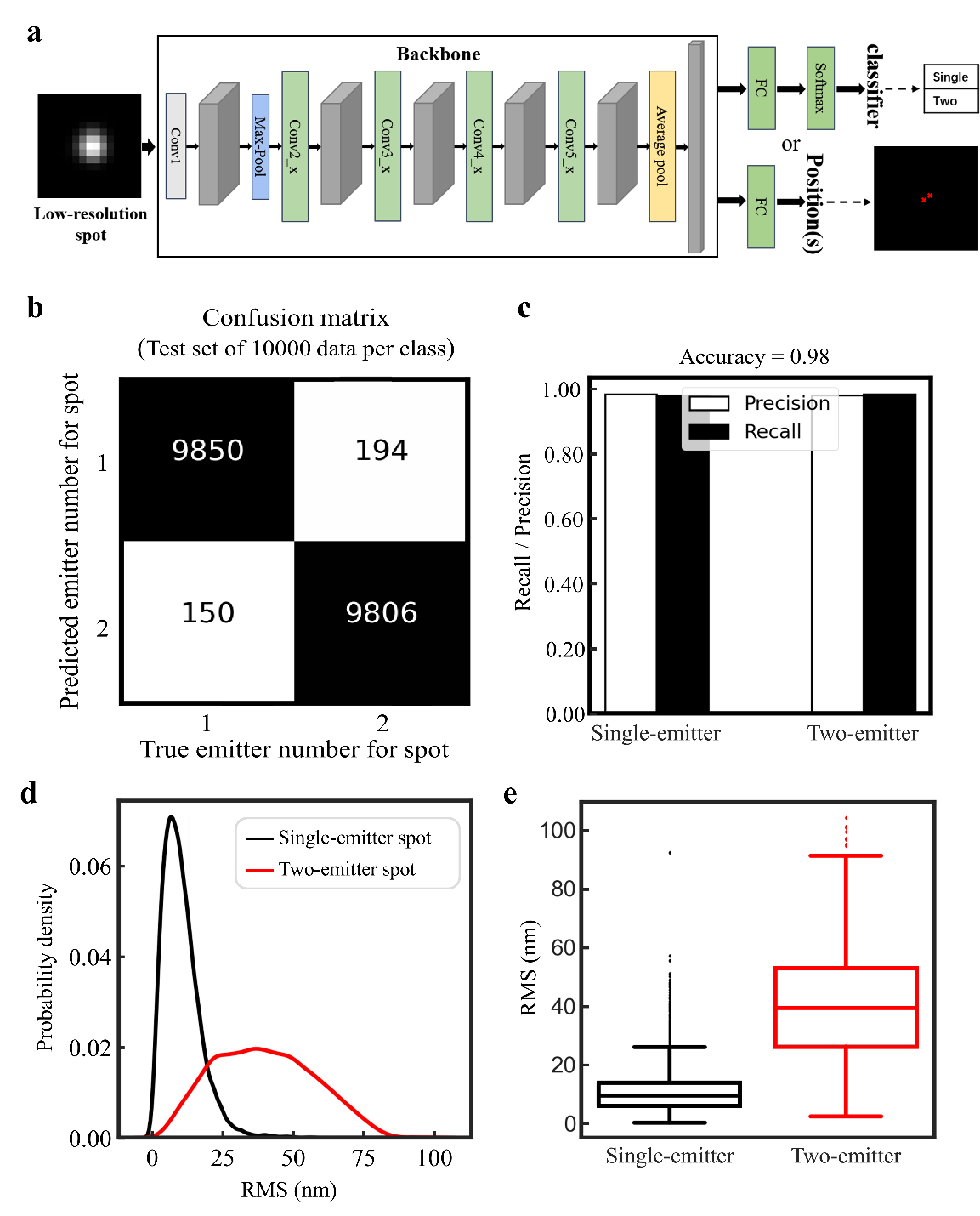


**Extended Fig. 4. An end-to-end network combining classification and localization and its performance experiments.** (a) We make a combination of the two-stage network mentioned in the text, by connecting the regression localization head and the classification head to the backbone at the same time, and the two heads with different functions do further processing on the features extracted from the backbone to accomplish the classification and localization functions respectively. (b) Confusion matrix plotted based on the prediction of the classification network on the test set, with the horizontal and vertical coordinates representing the number of true and predicted emitter number for spot, respectively. (c) Accuracy, recall and precision histograms. White bars show precision values for the varied spots, whereas black bars show recall. (d) The root mean square (RMS) values were calculated using the predicted coordinates and ground truth, and the probability density curves were plotted as shown in Fig. (e) The distribution box diagram of RMS is shown in Fig.


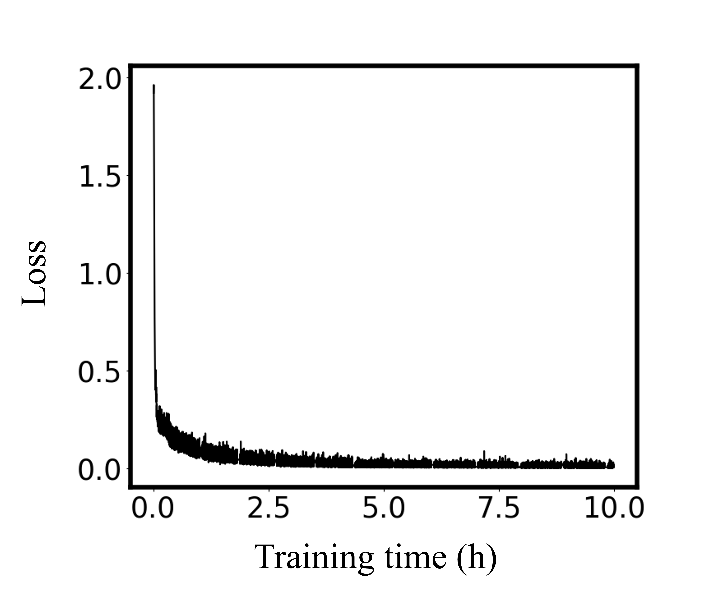


**Extended Fig. 5 Digital-SMLM loss change plot with time convergence.** This figure shows the convergence of the neural network model in the training process. The horizontal axis represents the training time, and the vertical axis represents the value of the network loss function. As the training progr esses, the network gradually learns and optimizes its parameters to minimize the loss function. The hardware specifications of the PCs used for training and testing in the above method are Intel 10-core, 12-thread,256GB of RAM, 2.2GHz cpu and NVIDIA Tesla V100S GPU (32GB RAM).

| Params | FLOPs | Forward/backward pass size (MB) | Params size (MB) | inference time (s) |
| --- | --- | --- | --- | --- |
| 23.511M | 0.320 G | 17.640 | 89.69 | 2.77 |

**Extended Table. 1 The number of network parameters, computational complexity, and inference time.**

References

[1] E. Nehme, L. E. Weiss, T. Michaeli, and Y. Shechtman, "Deep-STORM: super-resolution single-molecule microscopy by deep learning," *Optica,* vol. 5, no. 4, pp. 458-464, 2018/04/20 2018, doi: 10.1364/OPTICA.5.000458.

[2] N. Boyd, E. Jonas, H. Babcock, and B. Recht, "DeepLoco: fast 3D localization microscopy using neural networks," *BioRxiv,* p. 267096, 2018.

[3] R. Girshick, "Fast r-cnn," in *Proceedings of the IEEE international conference on computer vision*, 2015, pp. 1440-1448.

[4] S. Ren, K. He, R. Girshick, and J. Sun, "Faster r-cnn: Towards real-time object detection with region proposal networks," *Advances in neural information processing systems,* vol. 28, 2015.

[5] J. Redmon, S. Divvala, R. Girshick, and A. Farhadi, "You only look once: Unified, real-time object detection," in *Proceedings of the IEEE conference on computer vision and pattern recognition*, 2016, pp. 779-788.
